# Roosting behaviour of greater noctule bats (*Nyctalus lasiopterus*) in forests in Spain and implications for species conservation and forest management

**DOI:** 10.1098/rsos.251266

**Published:** 2025-08-06

**Authors:** Detlev H. Kelm, David Pastor-Bevia, Jesús Nogueras, Ana G. Popa-Lisseanu, Íñigo Sánchez, Carlos Ibáñez

**Affiliations:** ^1^ Ecology and Evolution, Estación Biológica de Doñana CSIC, Sevilla, Spain; ^2^ Evolutionary Ecology, Leibniz Institute for Zoo and Wildlife Research (IZW) in the Forschungsverbund Berlin eV, Berlin, Germany; ^3^ Zoobotánico Jerez, Jerez de la Frontera, Spain

**Keywords:** roost-switching, fission–fusion, roosting ecology, bat conservation, bat roosts, roosting area

## Abstract

The greater noctule (*Nyctalus lasiopterus*) is a threatened tree-roosting bat species with a fragmented distribution, possibly due to limited roosting habitat. Deforestation, tree disease and climate change are reducing forest and roost availability. Effective conservation action and forest management require detailed knowledge of the bats’ roosting behaviour and requirements, which is lacking for this species, particularly in southern European forests. We studied the roosting behaviour of 25 radio-tagged females from three maternity colonies in the forest and the urban environment, as well as 11 males from a forest mating site in Spain. We found similar behaviour and roost group sizes (14–18 individuals) for both sexes in the forest, where bats mainly roosted in woodpecker holes in larger trees of abundant tree species. Bats switched between many roosts (0.2–0.3 roosts d^−1^) across large forest areas (up to 1300 ha). At the urban site, females rarely switched between four exotic palm tree roosts, with roost group sizes reaching 144 individuals. Despite its adaptability, *N. lasiopterus* may require large forest roosting areas that provide a greater roost diversity, aiding thermoregulation and predator avoidance. Conservation efforts should focus on protecting large forests with high woodpecker abundance to ensure roost availability, supported by artificial bat roosts.

## 1. Background

Bats are a species-rich group of nocturnal mammals that require diurnal roosts to shelter during the day, and many species use tree holes in forests as roosts for all or part of their annual life cycle [1–3]. Roosts are also closely linked to the social behaviour in bats, and roosting conditions are an important aspect of their behavioural and physiological ecology [4–7]. Many temperate forest bat species prefer deciduous old-growth forests with deadwood due to their wealth of roost holes [3,8–10]. Forest composition has been found to have an effect on bat species occurrence (e.g. younger stands and intensively managed monocultures of coniferous species are less frequently used for roosting [10–12]). The presence of certain bat species (e.g. *Barbastella barbastella*) has even been proposed as an indicator of forest composition due to their preference for heterogeneous old-growth forests [13]. Therefore, the availability of suitable roost trees influences the distribution and abundance of bat species, making it a key consideration in bat conservation [3,9,14].

Forest conversion, loss and fragmentation affect bat species to different degrees. Some species are more flexible in their roosting requirements and use e.g. exotic tree species or anthropogenic roost structures, which enables them to persist following forest conversion and urbanization [5,15,16]. Mobile species, including open-space foragers of the genus *Nyctalus*, can cross an unfavourable matrix to occupy small and dispersed roosting habitats far from foraging habitats [5,16,17]. In contrast, bat species with small home ranges and specific habitat requirements, such as *Myotis bechsteini*, are negatively impacted by forest fragmentation and unfavourable management [18–21]. In addition, roost and habitat selection have been found to be sex-specific, also due to different energetic pressures and habitat requirements between sexes during reproduction [22,23]. Moreover, the quality of roosts and their thermal conditions have been found to influence the roosting behaviour, including roost group size and roost switching [24]. Consequently, to manage forests for the benefit of bats, it is necessary to understand the species- and sex-specific requirements of bats in terms of tree roosts and roosting habitats, taking into account regional habitat variability [14,22,25].

The greater noctule (*Nyctalus lasiopterus*) is a threatened insectivorous bat species that seasonally preys on migrating songbirds [26,27] and is probably one of the rarest bat species in Europe [28,29]. *Nyctalus lasiopterus* has a very large, but highly fragmented distribution range across Europe, from Portugal around the Mediterranean to the Urals, with the majority of the western European population occurring in Spain [28]. However, even within Spain, the species’s population is highly fragmented with very local occurrences, and stable colonies are known from only a few sites, mainly in larger forests in mountainous areas [30]. The reasons for this peculiar distribution pattern are not known, and it has been suggested that habitat quality, roost availability and the abundance of migratory bird prey influence the distribution of *N. lasiopterus* [10,28]. This species roosts in tree holes and seems to be associated with old-growth forest [10,28,31,32]. Therefore, anthropogenic habitat conversion and the loss of forest roosting habitat likely threaten the occurrence of this species.

Interestingly, the area with probably the highest abundance of the species and the largest stable maternity colonies was found in southern Spain, in areas surrounding the Guadalquivir river estuary, mostly in urban environments far from mature forests [28]. Popa-Lisseanu *et al.* [5,27] studied the roosting and social behaviour of *N. lasiopterus,* its diet and spatial movements, in a maternity population comprising three social groups totalling approximately 500 mostly female individuals that used more than 70 diurnal tree roosts, mainly in exotic tree species (e.g. *Platanus sp*.). The roosting site was a small 23 ha urban park in the centre of the Andalusian capital city of Seville (>600 000 inhabitants), which at the time was the largest known maternity population of *N. lasiopterus* [6]. Popa-Lisseanu *et al.* [5,33] found that females from maternity colonies form social groups that show fission–fusion behaviour [34], where the group splits into subgroups that disperse over different diurnal roosts. Members of a social group switch between roosts, changing the composition of the subgroups, but the social cohesion of the larger group remains stable over the long term, and females are highly faithful to individual roosts and to the roosting area (i.e. the area over which roosts are used) between years. *Nyctalus lasiopterus* has been found to have large home ranges of >1000 km^2^ and to forage seasonally up to 40 km away from their roosting area [33]. Another maternity colony has been found using artificial roost boxes in a small forest patch of roughly 2 ha in the mostly treeless floodplain of the Doñana National Park, approximately 40 km south of Seville [5,28,35,36] and another colony uses palms (*Washingtonia filifera*) in the city of Jerez de la Frontera as maternity roosts [26].

However, information on the roosting behaviour of *N. lasiopterus* in continuous forests in southern Spain is lacking, and there is little information on the behaviour of males, as previous studies have focused on maternity colonies, which are generally devoid of males due to sexual segregation for most of the year [37]. Although telemetry and observational studies of *N. lasiopterus* in forests in northern Spain [38], central Europe [39,40] and northern Italy [17] have generally confirmed the species’ previously described movements, home ranges and tree roosting, the datasets are often limited, and a comparison between sites in central and southern Europe is problematic due to the differences in climate and forest structure and tree species composition [41]. In addition, Popa-Lisseanu *et al.* [33] suspected that the peculiar situation of several maternity colonies in a small urban park with an abundance of roosts and a small isolated forest patch, where the bats used few roost boxes, affected the roosting behaviour and movements of the individuals in her study [26,28,33]. Consequently, it is unclear to what extent findings from studies in urban environments and from artificial roosts can be extrapolated to bats in forests in the same geographical region.

Efficient conservation action demands a detailed knowledge of the species’s roosting requirements in forests to enable population assessment and estimation, evaluation of habitat quality and informed decision-making for habitat restoration and forest management, including the installation of roost boxes. In addition, this information can contribute to the understanding of the species’ fragmented distribution and sexual segregation patterns.

The objectives of this study were to describe the roosting behaviour of male and female *N. lasiopterus* in continuous forest habitats in Spain, particularly with regard to roost group sizes and the number of roosts used over time, and to estimate the size of the roosting area during the reproductive period.

## 2. Methods

### 2.1. Study sites

**Figure 1.**
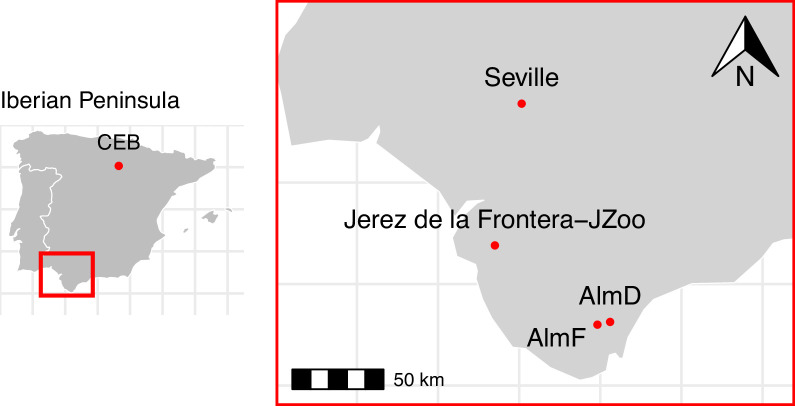
Location of the four study sites on the Iberian Peninsula. The city of Seville is indicated for orientation.

We studied the roosting behaviour of male and female *N. lasiopterus* in two different populations in Spain. We observed the roosting behaviour of females at three different sites in the province of Cádiz, Andalusia, in southern Spain (figure 1). Two of the study sites were located in the finca La Almoraima, near the town of Castellar de la Frontera (Cadiz), in the Alcornocales Nature Park, which holds the possibly largest cork oak forest on the Iberian Peninsula and one of the largest natural forests in the Mediterranean [42]. At the first site, ‘AlmF’ (100–365 m asl, 36.299° N, 5.516° W), the prevailing vegetation consists of a mostly Mediterranean oak forest, dominated by cork oak (*Quercus suber*), the deciduous oak species *Quercus canariensis*, strawberry tree (*Arbutus unedo*) and shrubland [41]. At the second site, ‘AlmD’ (25–118 m asl, 36.316° N, 5.435° W), the vegetation is more similar to a forest pasture (Spanish: ‘dehesa’) with intensively farmed cork oaks, *Q. canariensis* and cattle pasture. The mean monthly temperature in the Alcornocales Nature Park during the study period between May and August is 23°C (at 50 m asl; monthly *T*_min_: 16°, *T*_max_: 30°C), and mean monthly precipitation is 9 mm (see detailed meteorological information in electronic supplementary material, table S1). For comparison, we studied a third site (JZoo) in an urban environment in the Zoological and Botanical Garden of the city of Jerez de la Frontera (Province of Cadiz, 36.690° N, 6.150° W) with a park vegetation on 6.5 ha, including exotic trees, surrounded by urbanization and intensively farmed agricultural land. The average monthly temperatures and precipitation in Jerez during the study period are similar to those in the Alcornocales Nature Park (electronic supplementary material, table S1) [43]. The distance between the sites AlmF and AlmD is 7 km, and the distances between AlmF and AlmD and the Jerez Zoo (JZoo) are 72 km and 76 km, respectively. At all three sites, *N. lasiopterus* forms maternity colonies during the reproduction period between March and July, and male occurrence is scarce [37]. We collected the data at the forest sites between May and the beginning of August in the years 2011–2013, and at JZoo between mid-May and mid-July 2012.

We studied the roosting behaviour of male *N. lasiopterus* in the Sierra de Cebollera Nature Park (CEB) near the village of Villoslada de Cameros (Province of La Rioja) in northern Spain (42.065° N, 2.687° W; figure 1), where dense forests dominated by pine (*Pinus sylvestris*) and beech (*Fagus sylvatica*) alternate with mountain pastures and scrubland at altitudes between 1000 and 2164 m asl [41]. The mean monthly temperature in the Cebollera Nature Park during the study period between May and August is 15°C (at 1235 m asl.; monthly *T*_min_: 9°, *T*_max_: 21°C), and monthly mean precipitation is 46 mm (electronic supplementary material, table S1) [44]. Previous studies have shown that the Cebollera Nature Park is a key mating area for *N. lasiopterus*. The population consists mostly of males in spring and early summer, before the arrival of females of unknown origin (but unlikely from study sites of females described above) in mid-August, after maternity colonies have dissolved elsewhere [37]. At this site, we collected data between July and mid-August in 2013 and 2014. This site is located approximately 680 km north—northeast of the study sites of females, from where we had no information on male roosting areas during the reproduction period. In *N. lasiopterus*, the offspring are weaned between mid-July and mid-August, when maternity colonies dissolve and the behaviour in males and females may change towards the mating season [37]. Therefore, we did not include any behavioural data collected after mid-August in our study.

### 2.2. Data collection

We captured *N. lasiopterus* over creeks and at diurnal roosts with mist nets (100 D/2, mesh size 16 mm; Ecotone, Poland). We recorded the sex, age and biometric data of each individual and marked them with 5.2 mm aluminium alloy rings (Porzana, Icklesham, UK) on their forearms. Individuals were radio-tagged with transmitters (Pip Ag392; <2 g) attached to a Teflon collar, which was fixed to the back of the neck with skin glue (Manfred Sauer GmbH, Germany) [5], or with transmitters fixed directly to the back of the bat (Pip Ag376, 1 g; both Biotrack/Lotek, Dorset, UK), also using skin glue. The Teflon collar was designed to fall off after 1−3 months. We localized tagged individuals in their diurnal roost by homing-in on the transmitter signal using handheld three-element Yagi antennas (AF Antronics, IL, USA) and receivers (TRX-2000 and TRX-1000S; Wildlife Materials International, IL, USA). All individuals included in the study were localized on at least 9 days (i.e. not including two bats that lost their tags after a few localizations, and two bats that could not be found for unclear reasons after a few localizations). One individual was tracked in two years, and we discarded the second year of radiotracking for this individual.

We geo-referenced all diurnal bat roosts, identified the roost tree species, estimated the tree height and measured the diameter at breast height (DBH), the size of the roost hole, its height above ground and, if possible, the internal dimensions of the roost with a tape measure and a ruler. We also clarified the holes’ origin (e.g. woodpecker hole). To determine the roost group size, we conducted visual exit counts at roosts at dusk, either by direct observation or by video recording with infrared-sensitive video cameras (Sony HDR-PJ780VE, Japan) and an infrared spotlight (Raytec VAR-I2-1, UK).

### 2.3. Data analysis—roosting area, roosting group and roost switching

We estimated the land area on which diurnal roosts were used (‘roosting area’) both for individual bats and for a social group by calculating the convex hull for all roost locations in QGIS (v. 3.28.3) to determine a minimum convex polygon (MCP) [45]. In addition, we applied a kernel density estimation (KDE) to estimate the 50% and 95% core roosting areas of the colonies for which there was a suitable data set (AlmF, CEB) [46] using the R-package adehabitatHR (v. 0.4.21) [47]. To validate the estimation of a group’s roosting area, we plotted the MCPs over the chronologically incrementing number of localizations and distinct roosts of all radio-tagged bats in roosts [48]. To compare the size of the MCPs and the roost-switching rates between sites, we pooled data from all study years per site due to small sample sizes. We did not expect any significant variance in roosting behaviour, and we did not observe any changes in the habitat or roosts between the study years.

For the calculation of mean roost group sizes, we regarded repeated observations at the same roost with an observation interval of over 7 days as independent, due to the high roost-switching rate of *N. lasiopterus* [5,36]. We were not able to locate every bat on every day of the respective telemetry period (the time between tagging the individual and its last localization), either for logistical reasons or because we did not receive a transmitter signal. We defined the time before roost switching (i.e. roost-switching rate) as the time between the first day a bat used a roost and the day we did not record the bat in the same roost anymore, either because it had left the roost or because we were unable to locate it. Therefore, the roost-switching rates are maximum values, as the individual may not have switched roosts when we were unable to locate it. Exceptionally, in JZoo, because the bats rarely switched roosts, on four separate days when we were not able to use telemetry for logistical reasons, and the tracked individuals were found in the same roost as before, we assumed that the bats did not switch roosts on the days without telemetry.

We applied Kruskal–Wallis rank sum tests and post hoc Dunn’s pairwise tests with Bonferroni correction in R (v. 4.1.3) [49] to test for differences between the size of the MCPs. We applied two Kruskal–Wallis tests for the roost-switching rate and the number of roosts individual bats used per time, one comparing only the forest sites (AlmF, AlmD and CEB) and another also including the site in the urban environment in the zoo (JZoo), to assess the effect of the colony outside the forest. To test for the difference of the roosting group size between females and males in the forest habitat (AlmF, CEB), we applied a Wilcoxon–Mann–Whitney test.

### 2.4. Data analysis—roosting habitat and roost selection

To describe the bats’ roosting habitat and roost tree selection per site, we used data from the Spanish National Forest Inventory, which sampled trees with a DBH >75 mm on circular plots with a radius of 25 m on a 1 × 1 km grid in forested areas [41]. We pooled the data from plots adjacent to all roost trees at each site (mostly less than 1 km distance from roosts; electronic supplementary material, figures S1 and S2) as subsamples of the forest inventory and assessed the mean total tree cover, tree species abundance and tree DBH (the mean of two perpendicular measurements) per site.

We fitted a general linearized model with a binomial error structure in R [49] to model for each study site the incidence of roosts (dependent variable) as a function of tree size (DBH) and tree species and the interaction between these two independent variables. The dataset for the models included all study roost trees and all the trees in the national inventory subsamples of those tree species in which roosts were found. To validate the fit of the model, we tested the distribution of the residuals and the residuals against the predicted values using the DHARMa package (v. 0.4.6) in R [50].

## 3. Results

### 3.1. Roosting behaviour

**Figure 2.**
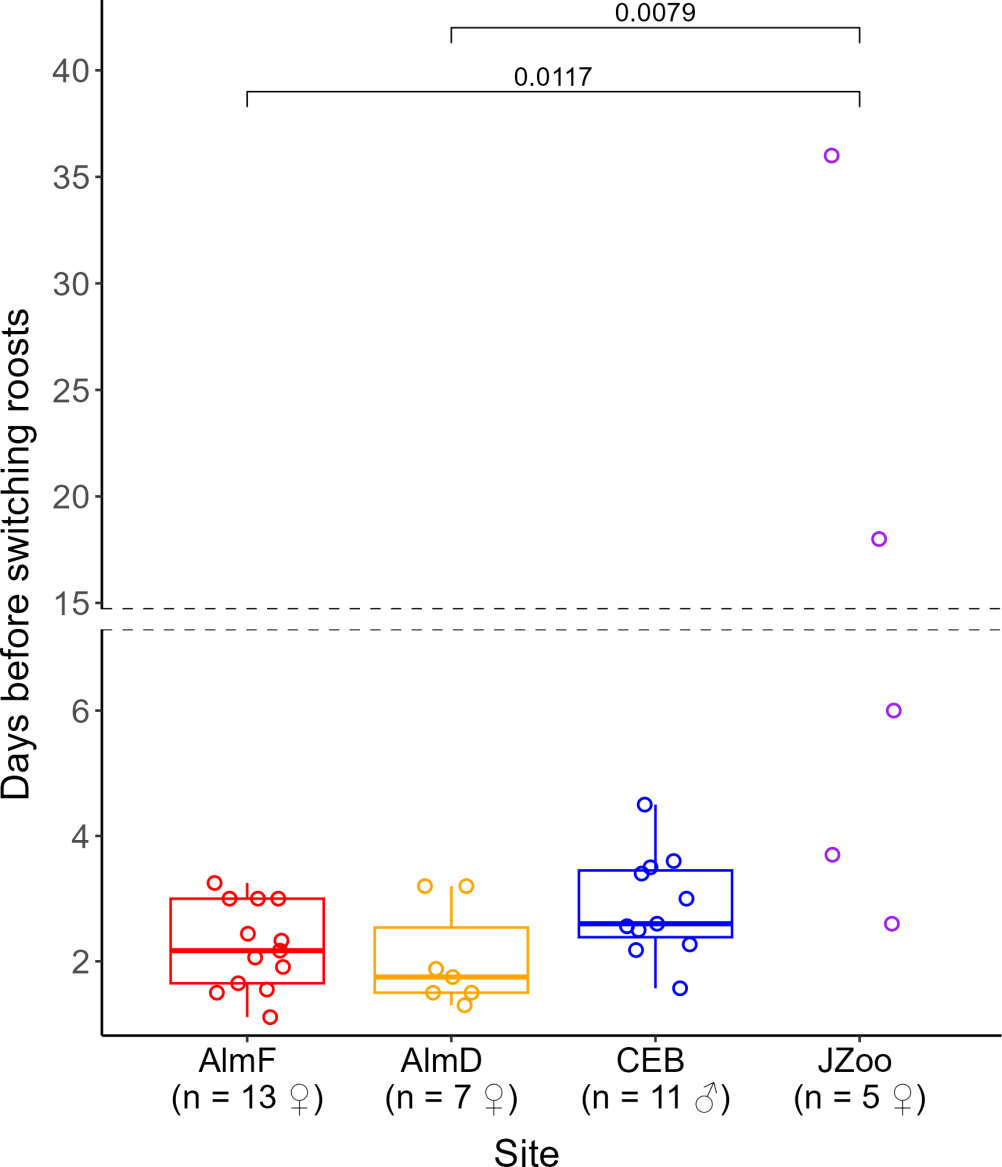
Roost switching (days before switching roosts) in *N. lasiopterus* between study sites. The lower and upper limits of the boxes represent the lower and upper quartiles (Q1 and Q3), the ends of the whiskers are the lower and upper limits of the data and the line in the boxes represents the median. The adjusted *p*-values of a Dunn’s pairwise test with Bonferroni correction are shown above the brackets for sites that differed significantly in roost-switching rates. At the sites AlmF, AlmD and JZoo, all tagged individuals were females. At CEB, all tagged individuals were males.

We radio-tracked 25 females and 11 males (table 1). All females were either pregnant or lactating at the time of capture. In the maternity forest sites (AlmF and AlmD), the average time between the tagging of a bat and the last localization (the telemetry period) was 42.4 ± 16.5 and 28.0 ± 6.5 days, respectively, and we located these females in the forest on an average of 49 and 48% of the days of the telemetry period, respectively, corresponding to 82 and 87% of the search days (the days that we searched for the bat during the telemetry period; table 1). Females in the urban environment at JZoo were located on average on 91% of the days of the telemetry period of 32.2 ± 5.3 days (i.e. 93% of search days). We located males in CEB on an average of 86% of all days of the respective telemetry periods (95% of search days), which averaged 22 ± 5.9 days per bat (table 1). The radio-tagged bats used a total of 30 roost trees in AlmF, 14 roost trees in AlmD, 48 roost trees in CEB and four roost trees in JZoo during the study periods. The roosting behaviour was similar between the forest sites (AlmF, AlmD and CEB). Bats switched roosts regularly from every 2.0 days in AlmD up to every 2.9 days in CEB (table 1), and there was no difference in roost switching between forest sites. A Kruskal–Wallis test indicated a difference between sites (Kruskal–Wallis chi-squared = 6.03, *df* = 2, *p* = 0.049), but this was not confirmed by the adjusted *p* of a post hoc Dunn’s test with Bonferroni correction. When the site JZoo was included in the comparison between sites, we found that the females in the zoo did not switch roosts as often (every 13.3 days) as females in the forest sites (Kruskal–Wallis chi-squared = 13.7, *df* = 3, *p* < 0.01; Dunn’s *Z* = 3.1, adjusted *p* < 0.03 and *Z* = 3.2, adjusted *p* < 0.01 respectively; figure 2). The number of different roosts used per day of telemetry was also lower in JZoo than in the forest sites AlmD and CEB (Kruskal–Wallis chi-squared = 13.1, *df* = 3, *p* < 0.01; Dunn’s *Z* = −3.2, adjusted *p* < 0.01 and *Z* = −3.3, adjusted *p* < 0.01, respectively; table 1). Females reused roost trees between years. In AlmF, the maternity colony used 41% of the roosts from the first study year (7 out of 17) in the following year. In AlmD, 2 years later, 25% (2/8) of the roosts were used. The male group used only 16% (5/31) of the roosts in the following year. During a total of 35 visual roost controls of 27 roosts in the forest habitat, we found no difference in the mean number of bats emerging from roosts between the roosting areas of females in AlmF and males in CEB (Wilcoxon–Mann–Whitney test: *Z* = 0.71, *p*‐value = 0.47). Roost group sizes varied, with 14.5 ± 12.3 bats in AlmF and 18.8 ± 14.4 bats in CEB, and the maximum roost group size was *ca* 50 individuals in both sites (table 1). Only in one case did we find a solitary bat exiting a roost in the maternity colony in the forest, and never in the male group. Due to the small sample size, we did not include AlmD in the test.

**Table 1.** Study effort per year and site, and different parameters of roost use in *N. lasiopterus*. Recaptures are individuals that have been captured in previous study years, and recaptures and juveniles are included in the total number of each year.

province	site	year	capture days (*n*)	captured		bats radio-tagged with locations on ≥9 days (*n*)	days localized per bat (mean ± s.d.)	different roosts used per bat and localized day (mean ± s.d.)	days before roost switch (mean ± s.d.) (range)	roosting group size (mean ± s.d.) (min/max)	number of roost trees used by tagged bats (*n*)	roost exit counts (*n*) /different roosts controlled (*n*)
				females (recap/juv.)	males (recap/juv.)							
Cadiz	AlmF (maternity colony)	2011	9	20 (0/0)	9 (0/1)	3	20.2 ± 6.9	0.24 ± 0.11	2.2 ± 0.7 (1.1 – 3.2)	14.5 ± 12.3 (1/51)	30	21/15
		2012	7	68 (13/10)	16 (1/7)	10						
	AlmD (maternity colony)	2011	2	7 (0/1)	1 (0/1)	2	13.3 ± 3.1	0.34 ± 0.15	2.0 ± 0.8 (1.3 – 3.2)	11.7 ± 6.5 (5/18)	14	3/3
		2013	3	8 (0/2)	0	5						
	JZoo (maternity colony)	2012	4	63 (0/6)	5(0/5)	5	29.4 ± 6.8	0.08 ± 0.05	13.3 ± 14.1 (2.6 – 36.0)	32.9 ± 42.9 (1/144)	4	12/6
La Rioja	CEB (mating site)	2013	3	0	10 (0/0)	4	18.9 ± 4.2	0.31 ± 0.08	2.9 ± 0.8 (1.6 – 4.5)	18.8 ± 14.4 (3/50)	48	11/9
		2014	8	0	46 (0/0)	7						

### 3.2. Roosting areas

**Figure 3.**
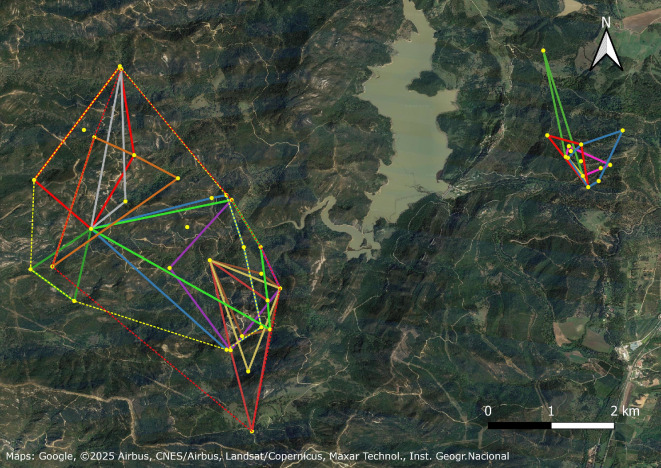
MCPs of the roosting areas of individual female *N. lasiopterus* in the Alcornocales Nature Park. Different colours represent different individuals. West is the site AlmF, and east is the site AlmD. The dotted yellow and red lines in AlmF are the MCPs of all roosts in 2011 and 2012, respectively. These are not shown for AlmD due to the small sample size. Southeast is the town of Castellar de la Frontera in the province of Cadiz, Spain (36.287° N**,** 5.420° W).

**Figure 4.**
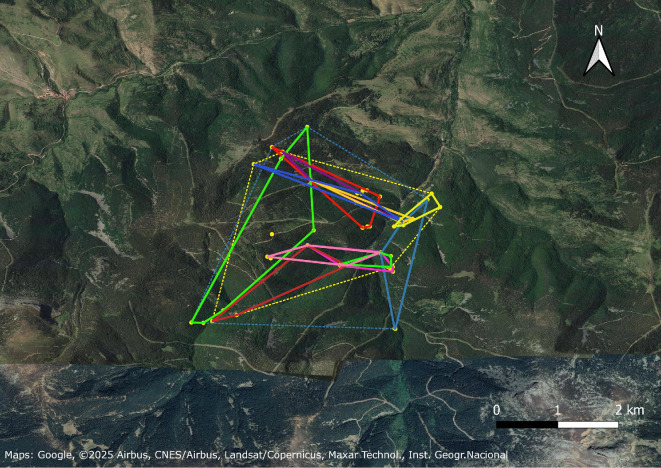
MCPs of the roosting areas of individual male *N. lasiopterus* in the Cebollera Nature Park, Spain (CEB). Different colours represent different individuals. The dotted blue and yellow lines are the MCP of all roosts in 2013 and 2014, respectively. To the north of the map is the town of Villoslada in the province of La Rioja, Spain (42.114° N, 2.674° W).

At the three forest sites, all radio-tagged bats had mostly overlapping roosting areas (described by MCPs of roost trees), and the individuals used partly the same roost trees, sometimes simultaneously (figures 3 and 4). In the maternity colonies (AlmF, AlmD and JZoo), all bats used roosts that were also used by at least one other bat in the study, sometimes simultaneously, and in AlmF, one roost was used by 8 of the 13 radio-tagged individuals over the course of the study (not simultaneously). However, none of the radio-tagged bats from any of the three female groups ever used a roost at another site, and none of the 183 marked bats was ever captured at another study site than the respective capture site (i.e. the sites’ roosting areas were clearly separated despite their proximity—7 km between AlmF and AlmD; figure 3).

**Figure 5.**
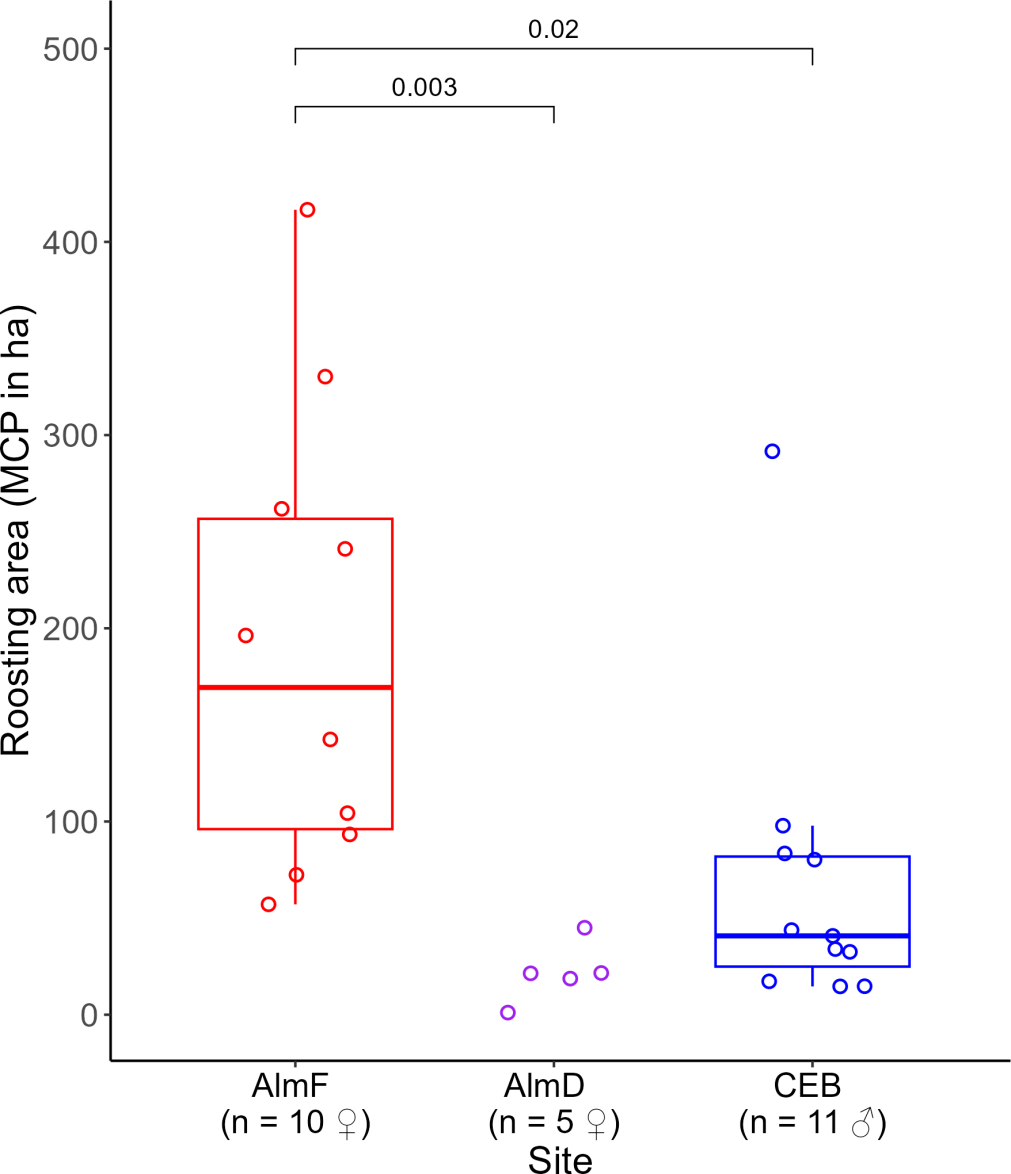
Roosting areas of individual *N. lasiopterus* (as MCPs in hectares) in different study sites. Adjusted *p*-values from a Dunn’s pairwise test with Bonferroni correction are shown above the brackets for sites with significantly different roosting areas. At both sites, AlmF and AlmD, all tagged individuals were females. At CEB, all tagged individuals were males.

The size of individual roosting areas (MCPs) was significantly larger in the maternity colony in the forest (mean MCP AlmF: 192 ± 120 ha, *n* = 10) compared to the roosting areas in the maternity colony in the forest pasture (mean MCP AlmD: 22 ± 16 ha, *n* = 5) and those of males in the mating site (mean MCP CEB: 68 ± 80 ha, *n* = 11; Kruskal–Wallis chi-square = 13.0, *df* = 2, *p* < 0.002; Dunn’s *Z* = 3.3, adjusted *p* < 0.01 and *Z* = 2.7, adjusted *p* < 0.02, respectively; figure 5). In AlmF and AlmD, MCPs could not be calculated for three and two individuals, respectively, as these bats only used two roosts during the telemetry study.

**Figure 6.**
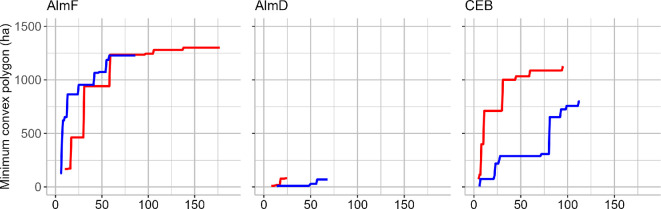
Incremental accumulation curves of MCPs (in ha) of roosting areas of radio-tagged *N. lasiopterus* in the forest sites with increasing number of localizations of bats in roosts during 2 years: AlmF (maternity colony, 13 females; blue: 2011, red: 2012), AlmD (maternity colony, 7 females, red: 2011, blue: 2013) and CEB (11 males, red: 2013, blue: 2014).

When plotting the accumulation curves of the area of MCPs of all roosts per site over the incrementing number of localizations of bats in roosts, we found that for AlmF (i.e. the site with the highest number of localizations; *n* = 86 and 177 in 2011 and 2012, respectively), the accumulation curve flattens at a roosting area of approximately 1200 ha after roughly 58 localizations in 2011 and 2012 with a maximum of 1228 at 58 localizations in 2011 and 1301 ha after 138 localizations in 2012, and a maximum distance of 3.45 km between roosts used by one individual. The estimates from a KDE are 2023 ha and 2113 ha for 95% of the roosting area in the two study years, respectively (table 2). At CEB, the accumulation curves for the roosting areas of all individuals did not seem to reach a plateau in either study year. The maximum roosting area was 1128 ha and 798 ha, and the KDE resulted in 2115 ha and 1314 ha in 2013 and 2014, respectively (table 2). However, the number of distinct roosts used within the MCP continuously increased at all sites (electronic supplementary material, figure S3). At AlmD, the sample size was too small to provide a verifiable roosting area of the roosting group (figure 6). At JZoo, the roosting area of the colony (approx. 0.5 ha) was restricted to the zoo, and the maximum distance between two roost trees was 153 m. Therefore, we did not include the zoo site in a test for the difference in the size of MCPs.

**Table 2.** Sizes of roosting areas of *N. lasiopterus* in two forest sites in Spain in different years, estimated as MCPs and with KDE for 50% and 95% of the roosting area. In AlmF, only females have been studied, while in CEB, only males have been studied.

site	year	MCP 100% **(ha)**	KDE 50% (ha)	KDE 95% (ha)
AlmF	2011	1228	513	2023
	2012	1301	486	2113
CEB	2013	1128	467	2115
	2014	798	270	1314

### 3.3. Roost characteristics

**Figure 7.**
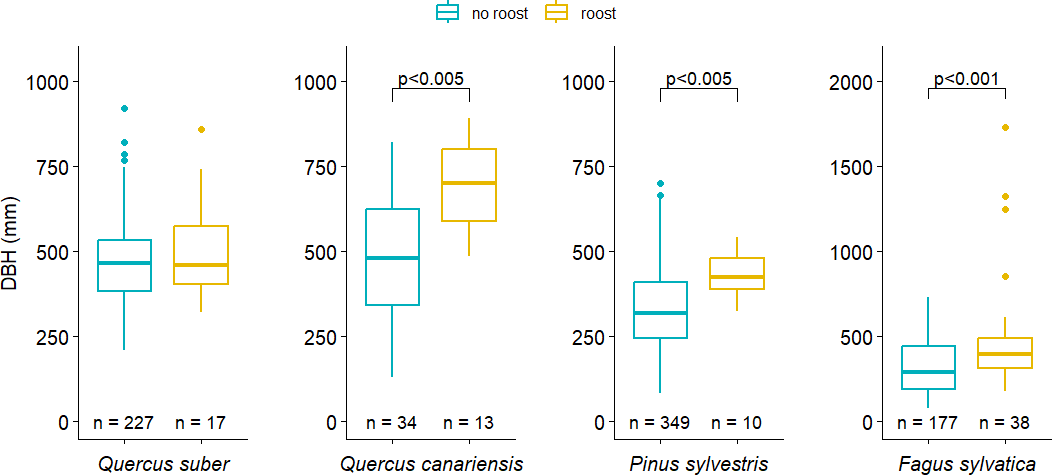
DBH of roost and non-roost trees of four tree species in the study sites AlmF (*Q. suber* and *Q. canariensis*) and CEB (*P. sylvestris* and *F. sylvatica*). Note the different scale of the *y*-axis for *F. sylvatica*. The *p*-values of Wilcoxon exact tests are shown above the brackets for species with a significantly different DBH between roost and non-roost trees (see also electronic supplementary material, table S3).

In all forest sites, roosts were mainly in woodpecker holes in larger, living individuals of the most common tree species. In AlmF and AlmD, we found all roosts in *Q. suber* (*n* = 27) and *Q. canariensis* (*n* = 17). *Quercus suber* contributed 53% of all trees (*n* = 429 trees) on the site’s selected plots of the Spanish Forestry Inventory [41] (electronic supplementary material, figure S1) and 40% of all trees (*n* = 47 trees) on the plots in AlmD. *Quercus canariensis* represented 8% of all trees on the forest plots in AlmF, but comprised 38% of all trees on the plots in AlmD. In CEB, all roosts were found in either *F. sylvatica* (*n* = 38) or *P. sylvestris* (*n* = 10). Here, *P. sylvestris* was the dominant tree species (58% of 602 trees on the selected forest plots), and *F. sylvatica* comprised 29% of all trees on the plots (electronic supplementary material, figure S2). In AlmF and CEB, 87% of the roost trees were alive, and at least 90% of the roosts in all tree species were in woodpecker holes (most likely of *Picus sharpei* and *Dendrocopos major*), except for *F. sylvatica*, where only 74% of all roosts were in woodpecker holes. Other roost holes were in broken branches and crevices in the tree bark. On the plots around roosts in AlmF and AlmD, the tree cover was similar at 55%, while it was 79% in CEB. When modelling the incidence of roosts in trees as a function of tree DBH and tree species, in both sites, AlmF and CEB, roosts were significantly more often in trees with a larger DBH (AlmF: *b* = 0.008, 95% confidence interval (CI; 0.003, 0.014), *p* = 0.004; CEB: *b* = 0.004, 95% CI (0.002, 0.006), *p* < 0.001; figure 7). In CEB, there was also a lower probability of roosts in *P. sylvestris* (*b* = −3.09, 95% CI (−5.72, −0.78), *p* = 0.013; electronic supplementary material, table S3). We did not further assess tree status and DBH in AlmD, due to the small roost sample size.

**Figure 8.**
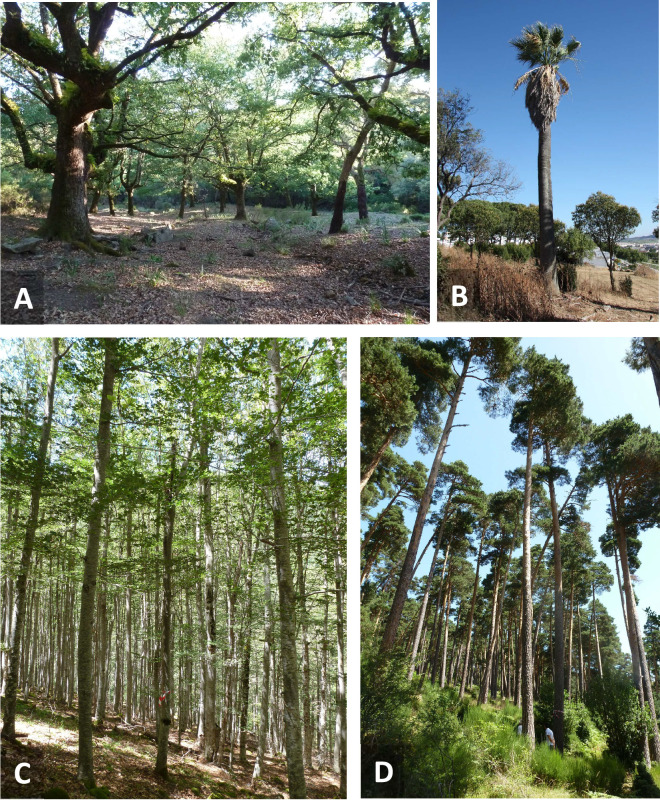
(A) Roosting habitat in the Alcornocales Nature Park (AlmF). (B) A roost palm tree (*W. filifera*) in the zoo of Jerez (JZoo). (C,D) Beech and pine forest in the Cebollera Nature Park (CEB) (photos: A,C,D: Detlev Kelm; B: Stefan Greif).

The mean height of roost cavities above ground in the different tree species ranged from 5.0 ± 1.2 m in *Q. suber* (*n* = 25) to 7.9 ± 2.3 m in *P. sylvestris* (*n* = 10). In *Quercus sp*., the roost entry holes had a mean diameter of 4.84 ± 0.65 cm (*n* = 16), an inner diameter of 28 ± 1 cm and an inner height above the entry of 85 ± 6 cm. We did not measure the inner dimensions of roosts in *F. sylvatica* and *P. sylvestris* in CEB. At JZoo, bats roosted under the dried fronds of four palm trees (*W. filifera*) at a height of *ca* 8−20 m (figure 8) [51].

## 4. Discussion

We found that the social groups of *N. lasiopterus* in the forest, both maternity colonies and male groups, disperse into subgroups and regularly switch between numerous roosts over remarkably large roosting areas (up to 1300 ha for one maternity group, calculated as MCP) during spring and early summer and reuse some of the roosts between years. This roost-switching behaviour generally conforms to the behaviour of a fission–fusion society that has been previously reported for maternity colonies of *N. lasiopterus* in an urban environment in the city park in Seville [5,6] and for other forest bat species [7,24,52]. However, the roosting areas in the forest were much larger than at our urban study site JZoo (0.5 ha) or those of maternity colonies in the city park in Seville (23 ha) [5], in mountain-forests in Slovakia (350 ha [33]; 670 ha [39,53]), and they are also much larger than roosting areas and home ranges of other forest bat species with fission–fusion behaviour [8,19,52,54].

The size of roosting areas may be determined by the abundance of *N. lasiopterus* and suitable roosts. We argue that the maternity groups studied (AlmF and AlmD) were separate, coherent, social units, as defined by Popa-Lisseanu *et al.* [5,33]. First, bats did not use roosts between sites despite their proximity (7 km) and the large distances *N. lasiopterus* can travel per night (up to 130 km when not migrating) [33]. Second, within groups, females shared roosts simultaneously and used common roosts. We argue that there was only one social group in each maternity forest site, as we also captured bats over water bodies for our study, thereby sampling bats independent of roosts and group membership. At the site AlmF, we captured 84 individuals of *N. lasiopterus* in 2012 and counted 69 individuals during simultaneous visual emergence counts at three roosts at the beginning of June, before the offspring had left the roost. Given the high fidelity of females to roosting sites during reproduction [5], we assume that this study group with a 1300 ha roosting area (MCP) contributed at least 5.3 adult roosting *N. lasiopterus* per km^2^ during the time of reproduction, and it may be a valid minimum density for the entire 566 km^2^ of oak forest habitats of the Alcornocales Nature Park [55]. For comparison, Popa-Lisseanu *et al.* [5] estimated approximately 500 individuals in three social groups in the 23 ha city park of Seville. We did not estimate the roosting area and group size at AlmD due to the insufficient sample size, as indicated by the insufficient plateau of the accumulation curves for the MCPs (figure 6), although the preliminary data show that the roosting behaviour did not differ much between the two sites, AlmF and AlmD (table 1).

Our study clearly highlights the dependence of *N. lasiopterus* on the presence of woodpeckers in the forest habitat, as bats roosted mainly in woodpecker holes, in the larger individuals of the most common tree species. In the sibling species *N. noctula*, other studies also found a high proportion of roosts in woodpecker holes [56], but comparisons are difficult as roost selection seems to be site-dependent. In an old-growth forest in Poland, *N. noctula,* and in particular *N. leisleri*, were more often found roosting in crevices of non-woodpecker origin [57]. In our study, possibly the larger body size of *N. lasiopterus* may limit the choice of roost crevices available to them. Although the height of the roosts selected by *N. lasiopterus* in our study roughly compares to that in other studies of *N. lasiopterus* [53] and of other *Nyctalus* species elsewhere [56,57], comparisons are difficult, as roost availability depends strongly on site characteristics, including tree species composition.

In our study, the selection of roost trees thus seems to reflect the selection of nest trees by woodpeckers, which have been shown to prefer nest trees with a softer heartwood (and possibly harder sapwood) [58–61], and tend to excavate holes in wood defects (e.g. broken limbs and heart rot) with soft substrate [62–65], conditions that may be more common in older, larger trees. As we lack information on the abundance of woodpeckers and their holes, we cannot assess the extent to which the size of the roosting areas is determined by the availability of suitable holes. However, we are not aware of any factor that would result in a lower roost availability than in similar forest habitats elsewhere, and suitably sized trees appeared to be abundant; for example, there were approximately 43 *Q. suber* ha^−1^ on the selected forest plots in AlmF, and 88% of all *Q. suber* in the plots (*n* = 227) had a DBH greater than the minimum DBH of a roost tree (32 cm) [41]. Consequently, the size of the social group in AlmF is likely to also depend on other environmental factors (e.g. food availability or predation). In CEB, bat roosts were significantly more frequent in *F. sylvatica* than in *P. sylvestris*, despite a higher abundance of pines and a higher wood density in *F. sylvatica* compared to *P. sylvestris* (0.69 and 0.55 g cm^−3^, respectively) [41]. This could indicate a preference of the bats for *F. sylvatica* and/or the associated forest type, or simply a lower incidence of woodpecker holes in pines. In this context, the comparable size of roosting areas of females and males in the forest is remarkable, as the tree cover in the roosting area of males in CEB is much higher (78%) than that in the roosting area of females in AlmF (55%) [41]. However, we acknowledge that in AlmF, we were not able to localize all individuals on all days, while in CEB, we found the bats on a higher proportion of days of the telemetry period (86%). Therefore, the roosting areas in AlmF could have been larger, as individuals might have used additional roosts outside the calculated MCPs. We caution that the KDE method to calculate the colony roosting areas might be prone to overestimation, particularly in situations when the roosting habitat is not continuous.

Male and female social groups in the forest used a high number of roosts, which is supported by similar observations (e.g. in Slovakia [53]). The long-term maternity colony with only four roosts in the zoo of Jerez is exceptional, because the cavity roosting *N. lasiopterus* adapted to roosting under dried palm fronds of an exotic palm species [26,51], which, contrary to tree cavities, provide a much larger roosting space (at least 144 individuals in one palm during our study), and appear to suffice for a large maternity colony.

The roost-switching behaviour in the forest habitat was comparable to previous observations in the city park of Seville [5]. However, bats in our urban study site switched roosts less frequently, possibly because they occupied only four palm tree roosts, whereas bats in Seville used more than 70 roosts [5,33]. Although the roost-switching frequency might decrease with reduced roost availability [36], we caution that the roost-switching frequencies of females in the forest sites in our study may be somewhat overestimated due to the partly limited durations of consecutive localizations.

Frequent roost switching is thought to help maintain group cohesion and inter-individual associations and may also be due to different individual roosting strategies (e.g. depending on the physiological status and thermoregulatory requirements), and individual levels of information about roost availability and quality [5,34,52,66]. A greater roost availability would therefore be beneficial in providing the bats with a greater diversity of roosts with different conditions and supporting the organization of optimal roost group size (e.g. for thermoregulation [24,67]). Furthermore, as suggested by Kelm *et al.* [36], the dispersal of the group across many roosts and a larger geographical area may help to reduce predation risk if predators are attracted to roosts that are more frequently visited for longer periods of time. Indeed, in all study sites, including the Seville city park [5], we observed the tawny owl *Strix aluco*, a predator of *N. lasiopterus* [36], approaching roosts during the bats’ emergence (C.I. 2011–2013, personal observation).

Males showed a similar behaviour to that of females in the forest regarding roost switching, roost group size and the dispersal of the roost group over a large roosting area. We acknowledge that a direct comparison between males and females is difficult, due to the large geographical distance and the different environmental parameters between sites. Nevertheless, the finding of male roosting groups is noteworthy, as in bat species with sexual segregation, solitary roosting of males is most commonly observed [68]. During sexual segregation, males may move to sub-optimal habitats to avoid intra-specific competition with females [37]. To balance their energy budget, males may engage in torpor, which may be supported by solitary roosting [69]. In contrast, in particular reproductive females should avoid torpor, which slows offspring development [70–73], and instead engage in social thermoregulation in clusters of roosting groups to reduce heat loss and thermoregulatory costs [66,67,74–77]. However, in our study, females might not be particularly constrained by low temperatures due to high diurnal ambient summer temperatures in southern Spain. A similar scenario could explain sociality and larger roosting groups in male *N. lasiopterus* in northern Spain. To prevent slowing spermatogenesis during torpor, males may engage in social thermoregulation in response to energetic constraints [78,79]. Safi & Kerth [68] proposed that information transfer on spatially and temporally clumped occurrences of ephemeral insects is a driver of male sociality. However, there are no studies on the importance of ephemeral insects in the diet of male *N. lasiopterus* that support this hypothesis for the study species. Benefits of sociality in male *N. lasiopterus* when foraging on birds are also unlikely, as bird predation occurs only towards the beginning of the mating season, when male groups likely have dissolved [37].

## 5. Conclusions and implications for species conservation

Our study shows that the roosting habitat and roost availability influence the roosting behaviour of *N. lasiopterus* during the period of reproduction and that social groups of both sexes use many roosts over very large roosting areas in the forest habitat. Maternity colonies in urban environments and the use of roost boxes indicate some flexibility in the selection of roosting sites. The exceptional ability to commute over very long distances may help to colonize sites that are far from foraging areas [33], persist in environments that have become unfavourable, including deforested and anthropogenically altered landscapes, and to use small forest fragments for roosting [17]. However, urban colonies seem to be limited to a few cases in Spain, and it is unclear to what extent colonization of uncommon roost sites can be expected elsewhere [80]. In addition, small and fragmented roosting sites may be prone to catastrophic events and poor colony stability (e.g. due to excessive natural predation [36]).

Indeed, effective conservation measures for *N. lasiopterus* seem to be urgently needed. The species is listed as vulnerable in the global IUCN assessment (International Union for Conservation of Nature) and with a declining trend for Europe [29], and its population is highly disjunct [32]. In particular, wind energy development threatens the viability of the population in Spain [81,82] and possibly elsewhere. In addition, the largest known maternity colony worldwide in Seville is in steep decline due to competition for roost holes with exotic ring-necked parakeets (*Psittacula krameri*) [83,84]. Continued deforestation and the loss of roost trees due to tree diseases (e.g. from fungal infection by *Phytophthora cinnamomi* [85]), sanitary forestry measures and possibly the loss of larger roosting habitats due to climate change [32,86–89] contribute to reducing the viability of the species not only in Spain.

Given the occupation of large roosting areas by single social groups in the forest habitat, we argue that it is crucial for the conservation of *N. lasiopterus* that land management programmes should aim to maintain large old-growth forests, as these may provide more favourable roosting conditions (e.g. in respect to roost diversity, social behaviour, thermoregulation and predator avoidance) than fragmented sites [24,36]. At the same time, forest protection and management should focus on the protection of woodpeckers [90], which are keystone species that provide nesting sites for various cavity breeders, and they appear to be essential for the occurrence of *N. lasiopterus*. Moreover, the protection of trees with woodpecker holes is essential to provide potential bat roosts [17]. Clear instructions are required and have already been implemented in the timber industry in some regions, including the site CEB, which prevents the felling of trees of a certain diameter with cavities. However, in forests that are suffering from disease, including infection by *Phytophthora* sp., implementing phytosanitary measures while at the same time protecting particular trees can pose a conservation dilemma. In this context, the large-scale installation of artificial roost boxes may help to overcome low roost availability in suitable areas with a limited number of roost trees.

## Data Availability

We include further graphs supporting our results in the electronic supplementary material. The data supporting the results of this study are available from the OSF repository [91]. Supplementary material is available online [92].
